# Multi-sector stakeholder's perceptions of determinants of successful implementation of a pediatric weight management intervention

**DOI:** 10.3389/fpubh.2022.954063

**Published:** 2022-08-25

**Authors:** Alicia Persaud, Ines Castro, Meg Simione, Justin D. Smith, Giselle O'Connor, Mona Sharifi, Meghan Perkins, Shioban Torres, Elsie M. Taveras, Karen Kuhlthau, Lauren Fiechtner

**Affiliations:** ^1^Division of General Academic Pediatrics, Massachusetts General Hospital for Children, Boston, MA, United States; ^2^Division of Health Systems Innovation and Research, Department of Population Health Sciences, School of Medicine, Spencer Fox Eccles School of Medicine at the University of Utah, Salt Lake City, UT, United States; ^3^Section of General Pediatrics, Department of Pediatrics, Yale University School of Medicine, New Haven, CT, United States; ^4^Bureau of Community Health and Prevention, Massachusetts Department of Public Health, Boston, MA, United States; ^5^Department of Nutrition, Harvard T.H. Chan School of Public Health, Boston, MA, United States; ^6^Division of Gastroenterology and Nutrition, Massachusetts General Hospital for Children, Boston, MA, United States

**Keywords:** pediatric weight management, childhood obesity, implementation science, stakeholder engagement, obesity

## Abstract

**Background:**

Multi-sector stakeholder engagement is essential in the successful implementation, dissemination, and sustainability of pediatric weight management interventions (PWMI), particularly in low-income settings where sustainability relies on external policies and reimbursement. The objective of this study was to engage stakeholders (1) to inform the creation of the intervention with adaptations needed for a successful PWMI in a primary care and community setting and (2) to identify barriers and facilitators to implementation and dissemination.

**Methods:**

We sought to examine the perspectives of local, state, and national clinic and community stakeholders during the pre-implementation period of a two-arm, randomized trial of a Health Weight Clinic PWMI conducted in two health centers and a modified—Healthy Weight and Your Child PWMI at two local YMCAs that serve a predominantly lower income, Hispanic community. The Consolidated Framework for Implementation Research interview guide served as a template for the study but was modified to fit the PWMIs and the various professional roles. Interviews were transcribed and analyzed using the framework analysis approach and themes were linked to the CFIR domains and constructs.

**Results:**

Twenty-six stakeholders perceived the following as needed components of a PWMI: a formal curriculum with illustrative examples, a patient- and family-centered program, group visits, and high-quality multidisciplinary personnel. These findings led to the creation of a group visit curriculum, implementation trainings and cross-site collaborative technical assistance. Additionally, creating partnerships between community and clinical organizations, and addressing patient barriers and unmet social needs (i.e., transportation, food) were identified as facilitators to successful implementation. These results led to the creation of community resource guides, connections to community organizations, and screening and referring for unmet social needs. Perceived facilitators of dissemination included proving cost-effectiveness of the PWMI to inform insurance reimbursement for long-term sustainability. Therefore, we collected cost data and engaged with Medicaid officials to discuss reimbursement.

**Conclusion:**

Findings highlight the importance of engaging multi-sector stakeholders pre-implementation to ensure the components valued are included, ensuring the program minimizes barriers to participation, considering how staff training can improve implementation and how collected outcomes can inform sustainability and dissemination of PWMIs in clinic and community settings.

## Introduction

Childhood overweight and obesity prevalence remains at historically high levels particularly in lower income and Hispanic and Black communities, and the COVID-19 pandemic has further exacerbated these disparities (1–3). The United States Preventive Services Task Force (USPSTF) found sufficient evidence to support that screening and intensive behavioral pediatric weight management interventions (PWMIs) for obesity in children and adolescents can lead to reduction in BMI (4). However, identifying the most effective components of these interventions and how to create sustainable, reimbursable interventions in clinic and community setting remains a major gap in the literature.

Many factors contribute to the intractability of childhood obesity but there are promising ways of reducing overweight and obesity including multi-sector, comprehensive programs in the primary care setting and the communities where children and their families spend their time (5–7). Implementation science suggests that stakeholders must be involved in the design of the intervention to ensure that the end goal of dissemination in under-resourced settings is achievable (8). Attention must be given to relationships between the characteristics of the intervention, those of the local setting and the priorities of local, state and national decision-makers. Furthermore, the use of a comprehensive theoretical framework can help identify the factors that are predictive of implementation success or failure and highlight strategies to achieve a successful implementation (9). Understanding the perspectives of stakeholders that have the potential to inform policy change, reimbursement, and sustainability is essential for successful implementation, dissemination, and maintenance of these interventions.

The USPSTF has recognized that identifying the most effective components of PWMIs is a major gap in current research stating, “Further investigations to determine the specific effective components of behavioral interventions are needed” (10). This study seeks to address this gap by completing a formative qualitative assessment during the implementation preparation period (i.e., the months leading up to the start of delivery of PWMIs) to contextualize individual stakeholder perceptions into executable concepts that can be applied to other similar interventions and programs. This study engaged multi-sector stakeholders in the pre-implementation phase of a two-arm randomized controlled trial in a clinic and community setting predominantly among Hispanic children from families with lower incomes to (1) inform the intervention components and adaptations needed for a successful PWMI in primary care and in the community setting and (2) identify barriers and facilitators to inform implementation and future dissemination of the intervention.

## Methods

### Setting

During implementation preparation of the *Clinic and Community Approaches to Healthy Weight* trial, we interviewed stakeholders from Massachusetts, where the intervention was conducted and national collaborators who were eligible by the stakeholder engagement terms outlined below. During the twenty-four-month study period, we engaged the stakeholders in bimonthly cross-site calls and biannual advisory meetings.

The *Clinic and Community Approaches to Healthy Weight* trial, which is described in detail elsewhere (11, 12) was a randomized controlled trial in two communities in Massachusetts that serve a large population of Hispanic children from lower income households. Inclusion criteria included the child had overweight or obesity, defined as body mass index (BMI) ≥ 85th percentile for age and gender. The two-arm trial compared the effects of Healthy Weight Clinics (HWC) in two federally qualified health centers (FQHC) to a Modified Healthy Weight and Your Child (M-HWYC) program delivered in the two local YMCAs among children 6 to 12 years old with overweight or obesity. In both communities, the participant population was predominantly Hispanic (93%), 69% of families made ≤ $20,000 per year, and 44 % of parents had less than a high school degree.

### Participants

We invited and interviewed 20 multi-sector stakeholders including pediatricians, dietitians, community health workers, behavioral health professionals, program managers, chief medical officers, local YMCA directors, state community health center representatives, national YMCA representatives, a Medicaid official, and a parent and patient who had participated in previous PWMIs (Table 1). To inform scalability and sustainability beyond this RCT, representatives (pediatricians, family medicine physicians and chief medical officers) from an additional six non-implementation health centers were chosen at random by the Massachusetts League of Community Health Centers to be interviewed. The individuals interviewed at these sites were those most familiar with the pediatric obesity efforts occurring in their practice setting. These interviews occurred during the first year of implementation (summer/fall of 2017). The non-implementation health centers varied in terms of the populations they served including differing racial/ethnic groups, and urban vs. rural populations. We recruited interviewees *via* email or through intermediary collaborators of the study *via* a snowball sampling approach.

**Table 1:** Participants in the Stakeholder Clinic and Community Approaches to Healthy Weight study (MA-CORD 2.0) qualitative interviews.

**Intervention site stakeholders (*N* = 20)**	***N***
Pediatrician	3
Dietitian	2
Community health worker	2
Behavioral health professional	2
Health clinic program manager	1
Patient advisor	1
Parent advisor	1
Local YMCA program director	2
MassLeague of Community Health Centers Representatives	2
National YMCA representatives	3
Medicaid official	1
**Non-implementation site stakeholders (*****N*** = **6)**	
Pediatrician	3
Family Medicine Physician	1
Internal Medicine Physician /Chief Medical Officer	1
Family Medicine Physician/ Chief Medical Officer	1

### Interview guide

Our interview guide consisted of questions related to stakeholder's views of effective intervention components and determinants to implementation and dissemination. The Consolidated Framework for Implementation Research (CFIR) (13) interview guide served as a template for this study with questions related to *Intervention Characteristics*: *Relative Advantage, Adaptability* and *Outer Setting: Cosmopolitanism, External Policies, and Incentives, Patient Needs and Resources* (Table 2).

**Table 2:** Interview guide based on CFIR constructs.

**Construct**	**Aim**	**Questions**	**Probes/Follow-up Questions**
**Intervention characteristics**			
Relative advantage	Feasibility of past pediatric weight management strategies in their health centers and community	We'd like to talk about your community's experience in the past with weight management programs and obesity treatment programs.	
		What has been tried in the past? In what setting?	
		What has worked and why?	
		What has failed and why?	
	Gaps and successes in past and current childhood obesity control efforts	What are the key elements to run a successful obesity program?	Specific program elements (advise about nutrition, cooking, portion size, physical activity…)
		How frequently should patients be engaged in the program?	Personnel?
		Do you have recommendations for resources/programs we should work with in your community or in the state?	If coaching is a priority who could deliver this? Community Health Worker? Registered Dietician? What would be ideal?
			Funding?
			Insurance Reimbursement?
			What are the most effective behavioral strategies in your opinion?
Adaptability	Preferred settings for pediatric weight management	We'd like to talk about the ideal setting for children and families to receive obesity treatment. In your opinion what would be the ideal setting for children to receive obesity treatment?	Community vs. Clinical: School, Home, YMCA, PWMI
			What makes this a good setting?
			Thoughts on using telephone-based, video-based or other technologies
**Domain 2: Outer Setting**			
Patient needs and resources	Major factors contributing to childhood obesity in their communities	What do you think are a few of the main contributors to childhood obesity in your community?	Lack of access to clinical care?
			Access to community resources such a physical activity, food?
			Poverty?
			Crime?
External policy and incentives	What would a pediatric weight management treatment package look like that would be appealing to payers	What would a childhood obesity treatment package look like that would be appealing to payers?	Private Insurance, Medicaid, examples of packages previously funded by payers i.e., Diabetes Prevention Program at the YMCA

### Interview procedure

Four researchers (LF, CH, GO, and KK) conducted the interviews by phone using the interview guide previously discussed. To ensure consistency and depth, two interviewers were present during all interviews. Informed consent and permission to have the interviews audio recorded was obtained. The interviews lasted ~30–45 mins and stakeholders were given $50 as remuneration. The Massachusetts Department of Public Health's institutional review board reviewed and approved all procedures.

### Data analysis

Interviews were transcribed and coded using the framework analysis approach (14). Interviews were audio recorded and transcribed by a professional transcription company (Landmark Associates). We uploaded the transcribed interviews into NVivo QRS 10.0 (QRS International Pty Ltd, Doncaster, Victoria, Australia) for analysis. Two interviewers (CH and GO) read each transcript independently to create inductively create codes from the source material based on the interview guide questions. The double coded interviews were compared in a tabular representation of the data to assure concurrence between the two coders. All researchers discussed discrepancies and agreed upon a final coding table by the entire research team through regular team meetings. All research team members convened in larger meetings to review the session content, coding, and emergence of themes which were linked to the CFIR domains and constructs. We chose direct quotes from the transcribed interviews to illustrate the findings (Table 3). Data analysis focused on the main interview topics, with a tailored focus for each interviewee's professional background.

**Table 3:** Illustrative quotes from stakeholders.

**Intervention characteristics**	
Design quality and packaging	
1. A formal curriculum with illustrative examples of healthy behavior change	
1a	“We eliminated [soda]completely because [the dietitian] had sugar in little bottles. The portions of sugar in bottles, how much sugar sodas have, how they harm us.”—*Parent Adviser*
1b	“Not just pointing pictures at the book but having the physical food there and the key was, portion control, so seeing what a plate looked like.”—*Patient Adviser*
1c	“I think the cooking demonstrations, also have to be about the recipes that would fit into what people are used to in terms of their heritage.”—*Pediatrician*
Adaptability	
2. A patient-centered program with a tailored approach	
2a	“Do this, do this, do this. Go home and eat this. Don't eat that.” They think that's what our program is going to be. It's not…Its behavior change model, which is “What do you think you can do?”—*Local YMCA program director*
2b	“[What] I think is a key element to be successful, because if the patient[s] do not feel comfortable with the providers they…will go to listen to you or they [don't make] many changes.”—*Behavioral Health Professional*
2c	“We've really had to learn a lot in this first session of, like, we sort of had the child sitting with their parent, and as we're facilitating the first hour, what we found was that the parents were doing all the talking…”—*Local YMCA program director*
Relative advantage	
3.3. A family-centered program where all members of the family are involved in behavior change	
3a	“Certainly, one of the things that was abundantly clear to me …that you cannot just do a program to change youth obesity with just the children. That's just never going to work. It can't work because …it's a family issue.”—*Local YMCA program director*
3b	“…one thing that is very, very helpful is pay attention to the interest of the family and support [and] connect the families with family partners or community support.”—*Behavioral health professional*
3c	“So, I think that there needs to be more focus on the parents and educating them because they're coming from a family, you know, they're in the same situation. So, some parenting skills, limit setting, cooking, shopping, and menu planning.”—*Dietitian*
4. Group visits to help build a support system for participants	
4a	“I think that group visits work better than individual visits …because of the support system…They don't feel like…they're the only ones. They have… other kids with them that are going through the same things.”—*Community Health Worker*
4b	“I think the special sauce is the relationships that they build with each other- and then sort of they feel responsible to each other, right?”—*Local YMCA Program Director*
4c	“I would've definitely wanted, being in a group setting. Especially [with] kids…around my own age; so, you can relate to them a lot…”—*Patient Adviser*
**Characteristics of individuals**	
Other personal attributes	
5. The inclusion of high-quality core personnel such as a community health worker, a physician, a behavioral health clinician and a dietitian	
5a	“I would say, number one, having someone who really knows the community and knows the culture of our patients…. Because if you can't understand our culture and our community, then whoever tries to teach is not going to get any parent to do anything.”—*Community Health Worker*
5b	“Be compassionate with people because some of the patients, especially the parents sometimes they come with long faces because of different issues.”—*Dietitian*
**Outer setting**	
Cosmopolitanism	
6. Partnerships of clinic and community organizations	
6a	“Neither one of us can do this work alone… clinical needs us, and we need clinical. Whether the partnership is around a referral source, or if it's …collaborative programming. I think we need each other”—*Local YMCA Program Director*
6b	“I think, to make it work better especially in my community, either the schools after school when you find a place like the nurse's office where the doctor can travel to, the different neighborhoods in the community, to access more people.”—*Patient Adviser*
6c	“I think with this new wave of quality improvement, and controlling costs…that might be the next phase where we…establish firmer relationships with effective community resources of the YMCA.”—*Internal Medicine Physician /Chief Medical Officer*
External policies and incentives	
7. Sustained funding for the program with insurance reimbursement	
7a	“I think it's very important that health insurance be providing reimbursement… it would create a priority, for different organizations to provide these services. If they can't find the funding, they won't be able to put more into it.”—*Patient Adviser*
7b	“It's going to be critically important, obviously, to manage chronic diseases as inexpensively as possible, and certainly it is cheaper to have a community health worker touch base with a family than it is to have a nurse or to have, you know, the provider… ACO models are probably going to incorporate more aggressive case management and we'll probably utilize … community health workers down the road. And so, I think that insurers will pay attention that because I think they're an inexpensive way to kind of in a culturally appropriate, linguistically appropriate way, to have health-related education to people who have chronic diseases.”*—Pediatrician*
7c	“I think it is important, and making sure it's evidence-based, which I think goes part and parcel with the cost …efficiency and quality equation, but then, going that step further to say, “Let's not look at it as a one-year, how much did you save,” but in the long run”*—Medicaid Official*
Patient needs and resources	
8. Identifying and developing solutions to patient barriers	
*Transportation*	
8a	“It's tough I think for them to get here, for families to actually come get into the clinic…They might take the bus, which is just a lot for them….They might not have the money to get here”*—Dietitian*
*Childcare*	
8b	“With the families having a lot of children, and lack of babysitters, they're coming here and having to bring them all…”—*Community health worker*
Time constraints	
8c	“The only problem with the weight clinic, for me, personally, was just—it was very time-consuming for the patients…”*—Community Health Worker*
Tertiary care centers are often where programs are occurring	
8d	“So that access for those programs are an issue, and when you have a disease that has, you know, has a 40 percent prevalence rate in our community, there is no way that those patients can all be seen at tertiary care centers. It's just not possible.”*—Pediatrician*
Cost	
8e	“but I think you should do something like that, or to help with obesity, or free groups to do exercise, or for people that don't have resources like me.”*—Parent Adviser*
Language	
8f	“In a perfect world, we would have in-person interpreters for all these visits.” *Pediatrician*

## Results

### Interview themes

#### Intervention characteristics

##### Design quality and packaging

###### A formal curriculum with illustrative examples of healthy behavior change

Stakeholders perceived a formal curriculum with illustrative examples of healthy behavior change as a key to how the intervention should be presented and assembled. The patient and parent that were interviewed highlighted the need for concrete examples and tips, such as illustrating sugar content in sodas or juices, to help facilitate healthy behavior changes. For example, the parent advisor said “We eliminated [soda] completely because [the dietitian] had sugar in little bottles. The portions of sugar in bottles, how much sugar sodas have, how they harm us.” Hands on activities such as cooking demonstrations were well regarded by providers attempting to engage participants. This led to the development of a group curriculum for the HWC that embedded illustrative examples and was iteratively changed and tailored by each HWC site.

##### Adaptability

###### A patient-centered program with a tailored approach

Stakeholders emphasized the importance of a patient-centered program that included an individualized approach tailored to each participant. Medical providers identified the significance of families feeling comfortable with the providers to motivate change. This led the research team to develop a protocol to train all physicians and team staff on motivational interviewing, a patient-centered counseling method aimed at enhancing intrinsic motivation to change health behavior (15). Additionally, teams were trained through the Kognito Interactive “Change Talk,” an interactive role-play simulation developed in collaboration with American Academy of Pediatrics (AAP) (16).

##### Relative advantage

###### A family-centered program where all members of the family are involved in behavior change

All interviewed stakeholders agreed that PWMIs cannot exclude the family members that support a child's lifestyle. Healthy lifestyle changes were thought to be most successful if the entire family practiced them together. From a sustainability standpoint interviewees expressed that if PWMIs could show effectiveness for the parents/caregivers involved, then this would prove a return on investment for insurers sooner than for the child alone. This contributed to the research team's decision to measure parental/caregiver BMI in the intervention and to set goals directed at all members of the family. However, in our most recent iteration of the HWC model we have not had parent's measure their BMI due to potential stigma associated with this.

###### Group visits to help build a support system for participants

Conducting the PWMI in a group setting was a popular idea with interviewed stakeholders. One community health worker expressed doubts about this format, citing that some children might be shy in a group setting and that it could possibly lead to weight related shaming. However, most of the discussion around the group setting for a PWMI was positive. For example, a community health worker said “I think that group visits work better than individual visits …because of the support system…They don't feel like…they're the only ones. They have… other kids with them that are going through the same things.” Stakeholders cited the invaluable benefit of a support system from other group members struggling with overweight or obesity. Group visit attendance was highlighted and encouraged to maximizing intervention contact hours and effectiveness.

### Characteristics of individuals

#### Other personal attributes

##### The inclusion of high-quality core personnel such as a community health worker, a physician, a dietitian, and a behavioral health clinician

Stakeholders remarked on the importance of having a fully trained multidisciplinary team that worked together. Key personnel on the multidisciplinary team listed in the stakeholder interviews included: community health workers, a medical doctor, a dietitian, and a behavioral health clinician. The community health worker role was seen as key because they are familiar with the population and can demonstrate cultural sensitivity in their support of a family's setting and achieving goals. Stakeholders noted that compassion, commitment, cultural sensitivity, and empathy were important qualities needed for providers in the PWMI. These qualities were promoted and emphasized consistently through hiring of implementation staff, implementation trainings, and cross-site technical assistance calls.

### Outer setting

#### Cosmopolitanism

##### Partnerships of clinic and community organizations

Interviewed stakeholders highlighted the importance of partnerships between clinical and community resources for childhood obesity. A local YMCA program director said, “Neither one of us can do this work alone… clinical needs us, and we need clinical. Whether the partnership is around a referral source, or if it's …collaborative programming. I think we need each other.” A Medicaid official spoke of using community settings as much as possible, particularly in an accountable care model. Many stakeholders described schools as being an important partner as children spend much of their time there. To address the ongoing social needs and connect families to low-to-no-cost physical activity resources, we offered community resource guides and ensured continued collaboration between each health center and their local community partners including schools.

#### External policies and incentives

##### Sustained funding for the program with insurance reimbursement

All stakeholders felt that funding was critical. Sources of funding were discussed, including grants and insurance reimbursements. However, stakeholders felt that for programs to be sustained, health insurance needed to provide reimbursement. They recognized that without reimbursement these programs could not be a priority for the clinics and YMCAs. Finally, stakeholders recognized that demonstrating the programs were cost-saving was vital to achieving insurance reimbursement.

The Accountable Care Organization (ACO) was cited as a model that might allow for programs to be sustainable. For example, the community health worker role was viewed as crucial, but the community health worker visits are not reimbursed in Massachusetts. Stakeholders pointed to the ACO model as potentially having the flexibility to cover the salary cost of a community health worker. Covering this cost would be important in the economics of chronic disease management. To ensure ongoing knowledge of and consideration for the priorities of the ACO, we invited ACO representatives to participate in cross-site calls and accepted guidance on how to facilitate sustainable implementation of the intervention.

A Medicaid official pointed to the importance of changing policy to support long-term changes in care and implementing those changes with clear research and long-term cost analysis. They said, “I think it is important, and making sure it's evidence-based, which I think goes part and parcel with the cost …efficiency and quality equation, but then, going that step further to say, “Let's not look at it as a one-year, how much did you save,” but in the long run.” Many stakeholders reiterated the importance of showing evidence that the programs offered effective pediatric weight management treatment to support larger policy changes. To consider this and further evaluate the return on investment in participation of the program, we collected data on costs of the program, direct additional costs for the family (i.e., purchasing healthier food, paying for children to participate in physical activities), and examined parent BMI for a sooner return on investment.

##### Patient needs and resources

Stakeholders interviewed were cognizant of logistic issues in attending a PWMI. They cited transportation issues for the families, lack of childcare for their other children and the time commitment for the families, which often conflicts with work or school. Providers also mentioned that programs are often occurring in tertiary care centers: a setting that is not possible for all patients to attend. Many stakeholders spoke about cost as a barrier for joining community programs and highlighted the need for free programs. Language barriers were reported by both patients and providers as an impediment to delivering the program and to effective motivational interviewing. To address this feedback the PWMI staff created the option for evening and weekend appointments, ensured bi-lingual staff were integrated in each visit, and verified the completion of motivational interviewing training.

## Discussion

In this qualitative study engaging 26 multi-sector stakeholders, we explored: (1) intervention components and adaptations needed for a successful PWMI in primary care and in the community setting; and (2) perceived barriers and facilitators to implementation and future dissemination to inform which implementation strategies to use. Stakeholders identified the following as needed components of a PWMI: a formal curriculum with illustrative examples for patients, a patient and family-centered program, group visits, and involvement of high-quality core multidisciplinary personnel. Perceived outer setting facilitators of successful implementation and dissemination included creating partnerships among community and clinical organizations, sustained funding, supportive policies such as insurance reimbursement, and identifying and addressing individual patient barriers to participation.

The curricula containing concrete examples of healthy behavior change was noted by stakeholders as a critical aspect for PWMI's, along with the need for the program to be patient- and family-centered. Previous studies have indicated that patient-centered programs are desirable for both patients and clinicians and improve health-related outcomes (17, 18). Furthermore, there is a growing body of literature to support the sustainability and effectiveness of family-centered interventions for childhood obesity (19–22). Given that children spend most of their early life with their family, it is essential that family members are ready to not only support but also be involved with the lifestyle change their child is implementing.

A novel finding was the preference for group visits as this is not a typical clinical PWMIs structure. While the YMCA Healthy Weight and Your Child program was already structured in a group visit format, stakeholder feedback informed the integration of group visits in addition to the individual visits offered into the HWC. In the evidence review from the USPSTF childhood obesity guidelines, group visits contributed to higher contact hour interventions, which were most effective in reducing BMI (4). Stakeholders expressed that high-quality multidisciplinary personnel including a community health worker, a physician, a behavioral health clinician and a dietitian was an important aspect of the intervention, which has also been cited in the literature as a critical component for success in a PWMI (23, 24).

As found in our study, fostering partnerships between the clinic and community organizations has been cited as critical to the success of interventions (25, 26). These relationships allow for the inclusion of creative ideas and solutions to problems drawing on multiple resources across the community, and making the best use of limited resources. These partnerships also help to address unmet social needs, which are known barriers to family behavior change. Our data suggested that addressing unmet social needs and clinical aspects of obesity concurrently are critical to PWMIs success in engaging families.

To address the outer setting concerns related to needing policies and reimbursement to sustain the program, we continued to engage with stakeholders from Medicaid, the Massachusetts Department of Public Health, and representatives from the FQHCs to discuss reimbursement options for the program. Without insurance reimbursement for the cost of operating PMWIs, low-resourced settings will struggle to provide services to their patients that are consistent with the USPSTF recommendations; and those inequitably impacted by childhood obesity will continue to be denied the recommended treatment.

Our findings from this study, informed our discussions in our technical assistance calls which occurred every 2 weeks throughout the intervention with implementing staff and our stakeholder meetings. In addition, the group curriculum, provider training, how to form clinic-community partnerships, how to create a sustainable model and addressing social determinants of health and barriers to retention and engagement have been integrated into our current HWC package that was created in collaboration with the American Academy of Pediatrics Institute for Childhood Healthy Weight and funded by the Centers for Disease Control for national dissemination (27, 28). The program is now being implemented by eight health centers in Mississippi and Massachusetts.

### Strengths and limitations

Strengths of this study include engaging a wide range of stakeholders, with a focus on those who can impact sustainability and dissemination including local program leaders, healthcare providers, and state and national decision makers. This study contributes to the literature of using stakeholder engagement to develop priorities and refine interventions (29–32). However, this study also has limitations. Since many of the stakeholders had a particular interest in treating childhood obesity, their views may not represent all providers and stakeholders in other communities. This study occurred in two communities that serve a majority Hispanic population with lower incomes in Massachusetts and findings may not be generalizable to other areas of the country and other patient demographics.

## Conclusion

Findings highlight the importance of the following: engaging multi-sector stakeholders' pre-implementation in PWMIs to ensure components stakeholders value are included, ensuring the program alleviates barriers to participation, considering how staff training can improve implementation, and how collected outcomes can inform sustainability and dissemination with potential insurance reimbursement in mind.

## Data availability statement

The raw deidentified data supporting the conclusions of this article will be made available by the authors, after an analysis plan is presented to the principal investigator.

## Ethics statement

The studies involving human participants were reviewed and approved by the Massachusetts Department of Public Health's institutional review board reviewed and approved all procedures. The patients/participants provided their written informed consent to participate in this study.

## Author contributions

LF helped to draft the original manuscript, designed the study, analyzed and interpreted the data, and approved and edited the final manuscript. AP helped to draft the original manuscript, interpreted data, and approved and edited the final manuscript. MSi and JS helped to interpret and revise the manuscript and approved the final manuscript. IC, GO'C, MS, and KK helped to analyze and interpret the data, revised the manuscript, and approved the final manuscript. MP, ET, and ST helped to design the study, edited the manuscript, and approved the final submitted manuscript. All authors contributed to the article and approved the submitted version.

## Funding

This study was supported by the Centers for Disease Control and Prevention National Center for Chronic Disease Prevention and Health Promotion (Award No.: U18DP006259). LF was supported by Grant Number K12HS022986 from the Agency for Healthcare Research and Quality and a K23HD090222 from the Eunice Kennedy Shriver National Institute of Child Health and Human Development. MSh was supported by Grant K08 HS024332 from the Agency for Healthcare Research and Quality and R01HL151603 from the National Heart, Lung, and Blood Institute. ET was supported by Grant K24DK105989 from the National Institute of Diabetes and Digestive and Kidney Diseases and Grant K24HL159680 from the National Heart, Lung, and Blood Institute.

## Conflict of interest

The authors declare that the research was conducted in the absence of any commercial or financial relationships that could be construed as a potential conflict of interest.

## Publisher's note

All claims expressed in this article are solely those of the authors and do not necessarily represent those of their affiliated organizations, or those of the publisher, the editors and the reviewers. Any product that may be evaluated in this article, or claim that may be made by its manufacturer, is not guaranteed or endorsed by the publisher.

## Author disclaimer

The content is solely the responsibility of the authors and does not necessarily represent the official views of the Centers for Disease Control, Agency for Healthcare Research and Quality, and the National Institutes of Health, or any other funders.
